# Genome analysis through image processing with deep learning models

**DOI:** 10.1038/s10038-024-01275-0

**Published:** 2024-07-31

**Authors:** Yao-zhong Zhang, Seiya Imoto

**Affiliations:** Division of Health Medical Intelligence, Human Genome Center, the Institute of Medical Science, the University of Tokyo, 4-6-1 Shirokanedai, Minato-ku, Tokyo 108-8639 Japan

**Keywords:** Genome informatics, Genetics research

## Abstract

Genomic sequences are traditionally represented as strings of characters: A (adenine), C (cytosine), G (guanine), and T (thymine). However, an alternative approach involves depicting sequence-related information through image representations, such as Chaos Game Representation (CGR) and read pileup images. With rapid advancements in deep learning (DL) methods within computer vision and natural language processing, there is growing interest in applying image-based DL methods to genomic sequence analysis. These methods involve encoding genomic information as images or integrating spatial information from images into the analytical process. In this review, we summarize three typical applications that use image processing with DL models for genome analysis. We examine the utilization and advantages of these image-based approaches.

## Introduction

Genome analysis has traditionally been conducted through string analysis [1], involving processes such as alignment [2–5], assembly [6–10], and structural variation detection [11, 12]. With the rapid advancement of high-throughput sequencing technologies, genomic data are expanding significantly across various dimensions, including data quality (e.g., read depth and length) and diversity of biological contexts and samples. These advancements present computational challenges in analyzing the increasingly complex and diverse genomic data, particularly at the whole-genome scale.

Meanwhile, deep learning models, such as Convolutional Neural Networks (CNNs), originally developed for applications in computer vision [13], have become powerful tools with potential applications in genome analysis. Deep learning models facilitate the learning of data representations across multiple levels of abstraction and enable the discovery of intricate patterns within large datasets [14]. In this article, we focus on the approach that performs genome analysis through image processing using deep learning models. This approach represents genomic data as image or image-like tensors and utilizes image-based deep learning methods for genome analysis. In the following sections, we begin with data representations for image and genomic data, then revisit three typical applications utilizing this approach. These include converting genomic sequences into image representations, using image recognition algorithms to identify patterns in genomes, and integrating both image and gene expression profiles.

## Image, genome, and deep learning model

**Fig. 1:**
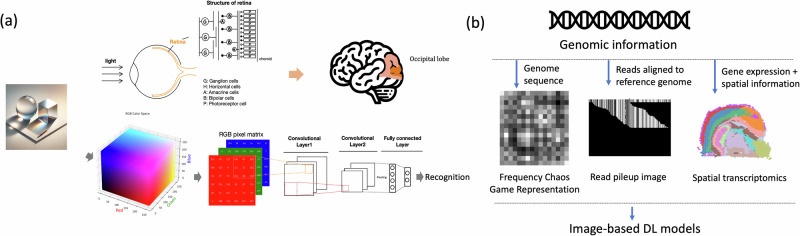
Overview of image processing by humans, computer systems, and genome analysis using image-based deep learning techniques. **a** The process of image processing by both humans and computer systems. In computer systems, colors defined by the RGB model are used for image representation and processed by a CNN model. **b** Three applications of genome analysis utilizing image processing with DL models, including genome representation, pattern extraction from read pileup images, and spatial transcriptomics. The pileup image is generated according to DeepVariant (r1.6). The spatial transcriptomics image is generated using the publicly available 10x Genomics Visium mouse brain dataset using squidpy package [80]

In the context of computer systems, an image is typically represented as a two-dimensional (2D) grid of pixels. Each pixel captures a portion of the visible light spectrum to recreate the appearance of a scene or object. The color and intensity of each pixel are quantified using color models, which define how colors are represented in the digital space. Common color models include RGB (Red, Green, and Blue), HSV/HSB (Hue, Saturation, and Value/Black), and CMYK (Cyan, Magenta, Yellow, and Key) [15–17]. The RGB model is one of the most common color models used in electronic displays, cameras, and scanners. It represents colors through a combination of red, green, and blue light, where the different intensities of these colors create the wide spectrum of colors that the human eye can perceive, as shown in the color cube of Fig. 1a. Consequently, image manipulations of filtering, transformation, and enhancement are performed based on the pixel matrix [18–20].

To the human eye, an image is perceived by the process of light being captured and focused by the eye’s optical system onto the retina. Cells in the retina convert the light into electrical signals and send them to the brain for processing and interpretation [21, 22], as shown in Fig. 1a. The retina is structured into three primary layers: the photoreceptor layer, the inner nuclear layer, and the ganglion cell layer [23]. Photoreceptor cells, known as rods and cones in the photoreceptor layer, convert the light into electrical signals [24]. Rods are responsible for vision in low light conditions and do not detect color, while cones are active in brighter light and enable color vision. The inner nuclear layer is located in the middle and contains several types of cells, including bipolar cells, horizontal cells, and amacrine cells. Bipolar cells receive signals from the photoreceptors and transmit them to the ganglion cells, acting as a direct relay [25]. Horizontal cells connect to multiple photoreceptor cells and are involved in integrating and regulating input to adjust for varying light levels, enhancing contrast. Amacrine cells interact with bipolar and ganglion cells, playing a key role in the timing and shaping of the signal as it passes through the retina. The integrated signals are then transmitted by the ganglion cells through their axons, which collectively form the optic nerve, to the occipital lobe of the brain [26].

Modern deep learning works by adding more layers and more units within a layer, providing a powerful framework for machine learning [27]. Deep learning models for images have been developed to train on large-scale image data [28–30] for various tasks, including classification [31], recognition [32], segmentation [33], and generation [34, 35]. Taking CNN [13] for instance, the success of AlexNet, a CNN-based model, in the ImageNet Large Scale Visual Recognition Challenge (ILSVRC) marked the beginning of an era dominated by modern deep learning models for image analysis [31]. This milestone in artificial intelligence demonstrated that CNNs could achieve remarkable accuracy in classifying and interpreting image data [36]. Convolutional layers, the core components of CNNs, operate through filters or kernels that detect specific features within an image, such as edges, textures, or shapes. This mechanism is analogous to the way the retina’s photoreceptors and subsequent neuronal layers respond to specific visual stimuli within their receptive fields. The structure similarity between CNN and retina is demonstrated in Fig. 1a. Based on the stacked convolutional layers, CNN can learn spatial hierarchies of features from input images.

Different from images represented in 2D-pixel matrices, genome sequences are typically represented as one-dimensional (1D) strings of characters. String-based algorithms are extensively used for tasks, such as alignment, searching, and comparison. BLAST (Basic Local Alignment Search Tool) [37] is an example of the string-based method. To leverage the capabilities of image-based deep learning models for analyzing genomic strings, two strategies are commonly used: one is adapting the architecture of models to process 1D sequences. For example, DeepBind adapts CNN for 1D sequence inputs to learn and predict protein-DNA/RNA binding preferences [38]. It utilizes one-hot encoding for nucleotide sequences, representing the nucleotides or k-mers as distinct binary vectors. The encoded sequence is then input into the tailored CNN model (e.g., the sliding window size is 4×*K*, where *K* is the window length) to learn and predict binding preferences. Through one-hot encoding, a 1D sequence can be transformed into a 2D matrix, but computational operations and pattern recognition are primarily performed across the sequence’s length. This is different from traditional 2D image processing, where operations are applied both horizontally and vertically across the image’s spatial dimensions. Distinguished from the above approach, the other is through the representation of genomic information as image or image-like tensors and using image-based deep learning models. Here, we focus on the latter approach for genome analysis, which has demonstrated promising results in recent works but is less systematically discussed.

## Genome analysis through image processing with deep learning models

### Converting genome sequences into images

Chaos Game Representation (CGR) [39] emerges as a powerful technique offering a way of portraying genome sequences through fractal images. Originating from the field of mathematical chaos theory, CGR translates the sequential information of a genome into a spatial context, enabling the visualization of nucleotide arrangements and their patterns across entire genomes. This transformation is achieved by representing nucleotides as fixed points in a multi-dimensional space and iteratively plotting the genomic sequence to unravel hidden structures. The efficacy of CGR lies in its ability to condense extensive genomic data into compact, visually interpretable images, preserving the sequential context and highlighting peculiarities that might be elusive in traditional sequential representations. As a result, CGR facilitates a holistic understanding of the genome’s structural intricacies, promoting the discovery of functional elements, genomic variations, and evolutionary relationships.

**Fig. 2:**
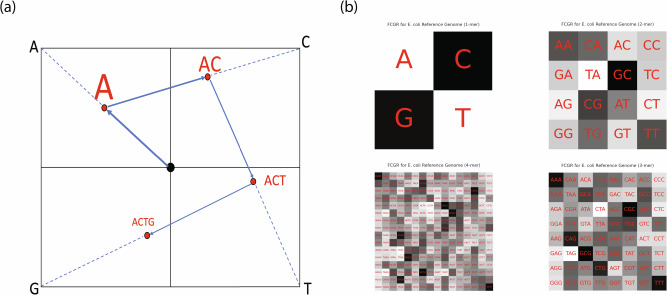
Frequency chaos game representation for genome sequences. **a** Position finding algorithm for chaos game representation: the algorithm starts at a fixed point (indicated by a black dot) and progresses to determine the positions for k-mers. These positions, represented by red dots, are calculated as the midpoint between the current position and the position corresponding to the nucleotide of the k-mer. The blue arrows illustrate the sequence of steps to locate the specific position of a k-mer in the FCGR image, with progression from 1-mer to 4-mer. **b** Frequency chaos game representation of the E. coli reference genome: this figure depicts the FCGRs for the E. coli reference genome across different k-mer lengths, ranging from 1-mer to 4-mer. The gradient from white to black signifies the frequency of each k-mer within the genome. The size of each box in the representation correlates with the resolution of the FCGR image or the length of the k-mer

The Frequency Chaos Game Representation (FCGR) extends the CGR by incorporating k-mer frequencies [40]. In FCGR, the bit depth of the CGR image encodes the frequency information of k-mers. Generating an FCGR image involves several key steps. Initially, the length of the k-mer is selected, which influences both the granularity and the complexity of the resulting FCGR image. This selection strikes a balance between resolution and computational efficiency, with longer k-mers providing more detail at the expense of increased computational requirements. After determining the k-mer length, the next step is to calculate the frequency of each k-mer within the target genome. This process involves scanning the entire genomic sequence to count the occurrences of each unique k-mer. These frequencies are then used to construct the FCGR image, where each k-mer corresponds to a specific pixel or a group of pixels. The placement of these pixels in the FCGR image follows the rules of the Chaos Game, which assigns each k-mer a unique location based on its nucleotide composition. This approach transforms the linear, sequential genomic information into a two-dimensional, spatial representation. The result is a fractal-like image that visually encodes the distribution and relationships of k-mers throughout the genome. As demonstrated in Fig. 2b, the visual complexity of the FCGR image increases with the k-mer length. More specifically, a 1-mer FCGR might appear relatively simple and uniform, whereas a 4-mer FCGR exhibits a more intricate and detailed pattern. This increase in resolution reveals more nuanced aspects of the genomic sequence, such as the prevalence of certain k-mers or the presence of repetitive elements.

As an image representation, the FCGR can be seamlessly integrated with image-based deep learning models for genome analysis [31, 41, 42]. Mill´an Arias et al. [43] explored the use of deep learning algorithms in conjunction with FCGR for genome clustering, which can effectively process DNA sequences over 1 billion bp and outperform two classic clustering methods (K-means + + and Gaussian Mixture Models) for unlabelled data. Hammad et al. [44] used a pre-trained convolution neural network to extract features from FCGR images and selected the most significant features for downstream classifiers for COVID-19 detection. Besides using deep learning models with FCGR representation, Zhang et al. proposed to use contrastive learning to further learn the representation that integrates phage-host interactions based on FCGR [45]. In addition, they conducted an ablation study to demonstrate the performance gains achieved through the 2D representation of the k-mer vectors used in conjunction with the CNN. Compared to the use of k-mer frequency features as a 1D vector, FCGR introduces an additional dimension by arranging k-mers such that higher-order features can be extracted. For example, k-mers sharing the same suffix are positioned in close proximity (e.g., 3-mers of {A/C/G/T}**AA** as illustrated in the top left corner of the 3-mer FCGR in Fig. 2(b)). Consequently, when applying a CNN to FCGR, the convolutional module can effectively extract features associated with k-mers that have identical suffixes. The synergy between FCGR and advanced deep learning methods potentially leads to more powerful tools for genome analysis.

Besides their application using deep learning models, CGR and FCGR can be employed with alignment-free algorithms for genome comparison and phylogenetic analysis [39, 46, 47]. For instance, Jeffrey demonstrated how CGR could be used to visually differentiate between the genomic sequences of various organisms, offering an effective approach to studying phylogenetic relationships [39]. Local and global patterns between genome sequences can be compared without alignment to reference genomes. The work by Deschavanne et al. [40] showed the effectiveness of using FCGR as signature patterns for genomic sequences, and the utility of image distances as a measure of phylogenetic proximity.

When comparing string-based and image-based methods, it is evident that each has its unique advantages and limitations. The string-based analysis is well-established and efficient for raw sequence data processing, but it may lack the intuitive insight provided by a visual representation. Image-based analysis, on the other hand, offers a more intuitive and detailed view of complex genomic patterns but requires more computational resources and expertise in image processing and deep learning. In practice, the choice between these methods depends on the specific requirements of the research. For tasks requiring direct manipulation and comparison of raw sequences, string-based methods are more suitable. Conversely, for applications where pattern recognition and complex relationship analysis are crucial, image-based methods may offer insights.

### Detecting patterns in read pileup images

**Fig. 3:**
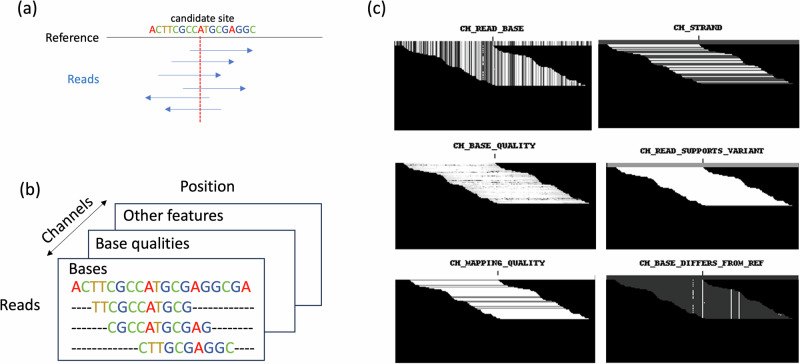
Read pileup images generated by DeepVariant. **a** Sequencing reads aligned to the reference genome. **b** Read pileup images derived from read alignment. **c** Pileup images of different feature channels generated by DeepVariant, with the example of Pileup chr20:10001627_*G*→*A* (GRCh38.p13 build) provided by DeepVariant

Image-based deep learning methods can be utilized in processing information derived from sequencing data. Genome analysis is a complex and multifaceted discipline that involves the interpretation of vast amounts of sequencing data to uncover genetic variations and understand their implications. To identify genomic variants from sequencing data, the first step is to map sequencing reads to the reference genome (Fig. 3a). Alignment information is crucial for detecting genomic variants such as single nucleotide variants (SNVs), insertions, deletions, and more complex structural changes [48].

Traditionally, read alignments and variant calling rely on text-based representations and statistical approaches [49, 50]. With the advent of deep learning methods in bioinformatics, there has been a paradigm shift towards more sophisticated and data-driven methodologies. One representative method is DeepVariant [51], which converts the read alignment data into read pileup images for variant calling. The read alignment information is transformed into a multi-channel image-like tensor, in which each channel corresponds to a specific aspect of the data, including features of read base, base quality, mapping quality, strand information, read support variants and base differs from the reference genome, as shown in Fig. 3b, c. The information can be equivalent to those shown in the IGV [52] that are commonly used in human experts’ manual evaluation of a putative variant. This image representation can capture the spatial relationships and patterns within the read alignments, providing a rich and contextual view of the candidate genomic region. For read pileup images, DeepVariant uses CNNs to predict the likelihood of different genotypes of homozygous reference (hom-ref), heterozygous (het), or homozygous alternate (hom-alt). The CNNs in DeepVariant are used to consider extensive contextual genomic alignment information, ensuring that the predictions are not solely based on the immediate read alignments. Additionally, these predictions are also influenced by the broader genomic context and overall aligned reads. More importantly, such a method can generalize well across different genomes and different sequencing platforms for variant calling [51, 53]. Not limited to the CNN model, other deep learning models, such as generative adversarial networks, have been applied for pileup image features [54]. Besides detecting SNVs and small-indel variants, extensions of read pileup images have been developed for calling structural variants (SVs) [55–57]. A recently proposed method named Cue [56] transforms read alignments to images for encoding SV abstractions and uses a specific CNN to predict SVs. Such a method does not rely on hand-crafted features, demonstrating enhanced generalization capabilities and competitive performance.

A similar image visualization technique has also been employed in nanopore sequencing. For nanopore basecalling, short-tandem repeats (STRs) tend to exhibit a higher error rate. To address this challenge, Fang et al. [58]. developed DeepRepeat, a method that transforms ionic current signals of reads into RGB channels for predicting repeats. More specifically, the current signals of STR and its upstream and downstream units are converted into RGB channels of a color image. In the image, the height and width represent signal range and STR unit size, respectively. Images from repeat and non-repeat regions are used to train a CNN model for identifying the presence of repeats. Their experimental results demonstrate that this approach can effectively detect STR regions, showing advantages over previous non-image-based methods.

### Spatial transcriptomics: integration of spatial image information and gene expression data

Different from the previous two applications that transform genomic information into image representations, gene expression profiles can be analyzed in conjunction with image data. Spatial transcriptomic (ST) integrates spatial information from tissue organization with gene expression profiles, which extends the ability for the genomic understanding of tissue organization, cellar interactions, and disease [59]. For instance, in cancer research, ST can reveal how different regions of a tumor may express distinct gene profiles, potentially leading to variations in treatment response and prognosis [60]. In neuroscience, ST can help in mapping the diverse gene expression patterns in different brain regions, offering insights into the molecular mechanisms underlying brain function and disorders [61]. The spatial dimension introduces an additional layer of information not available through traditional transcriptomic analysis.

According to different visualization and transcript detection methods, ST technologies can be classified into two main categories: imaging-based ST and sequencing-based ST. Imaging-based ST techniques, such as seqFISH [62], MERFISH [63], and ISS [64], employ fluorescence microscopy to probe specific transcripts within a slice at single-cellular resolution. Sequencing-based ST techniques, such as 10X Visium and Stereo-seq [65], capture the entire transcriptome at spot resolution through spatial barcoding. Currently, most sequencing-based ST platforms provide a spot resolution that contains 10 to 15 cells per spot [66].

Given the multi-modal nature of ST data and variability in techniques, analyzing raw ST data is far from trivial [67]. On the one hand, accurate processing of image and transcript data is essential to obtain spatial coordinates and gene expression profiles. On the other hand, integrating image and transcript data adds another layer of complexity. For imaging-based ST data analysis, image processing techniques such as correction, registration, and segmentation are routinely employed. These processing techniques are essential to enhance the quality and reliability of the data, facilitating more accurate mapping of gene expression within tissue sections [68]. Identifying cell boundaries is fraught with challenges due to the high variability in cell shapes, sizes, and densities, especially in heterogeneous tissue samples. Advanced image processing algorithms are required to overcome these challenges, ensuring precise and accurate segmentation. Even for ST techniques that require minimal image processing, additional steps are necessary after sequencing to map transcripts back to their spatial coordinates [69]. The accompanying tissue images can provide useful information for this mapping process, enhancing the accuracy and effectiveness of spatial localization. Upon acquiring a transcriptomically and spatially coherent mapping, the processed ST data can be transformed into image-like tensors for downstream analysis. Similar to RGB channels in an image, both color information and gene expression profiles can be encoded as channels to represent ST data. Deep learning models are effective at handling multi-modal data, making them suitable for analyzing both image and gene expression data in spatial transcriptomics.

Deep learning models have been employed in various applications, including cell/nuclei segmentation [70], alignment [71], spot deconvolution [72], and spatial clustering [73–75]. Various deep learning architectural frameworks, such as CNNs, autoencoders [76], U-nets [70], graph neural networks [77], and transformers [78] have been utilized either individually or in combination to address these challenges. For instance, Tangram [71] integrates molecular and anatomical features by combining a Siamese neural network with a segmentation U-net model to generate full segmentation masks of anatomical images. The U-net architecture is built based on a ResNet50 backbone. SpaCell [73] employs a pre-trained ResNet50 (trained on ImageNet) along with two separate autoencoders to generate a latent matrix representative for both image and gene-count data. SpaGCN [74] used a graph convolutional network for a constructed undirected weighted graph that integrates gene expression data, spatial location, and histological information for representing the spatial dependencies within the data. Chen et al. proposed sub-cellular spatial transcriptomics cell segmentation (SCS) to combine image data with sequencing data to improve cell segmentation accuracy based on a transformer model [66]. The proposed SCS method demonstrates a better performance when compared with traditional image-based segmentation methods.

## Discussion and concluding remarks

In this article, we reviewed the methodologies that conduct genome analysis through image processing with deep learning methods. The efficacy of image visualization techniques in genome analysis can be attributed to the effective information representation and the usage of advanced deep learning models. With the advancements in sequencing technologies, genomic profiling has become richer and more comprehensive. This expands the versatility of genomic data beyond the traditional string-based formats. Representing genomic information as image inputs provides a solution to represent various genomic data in 2D space with multiple channels. The image representation can be further processed with the advanced deep learning models for genome analysis. Furthermore, image processing is indispensable for processing image data in spatial transcriptomic analysis. Distinguished from the traditional one-hot encoding approach, using image-based deep learning models for genome analysis can be characterized by the addition of extra dimensions beyond the sequential level. Such an extension further leverages the capabilities of deep learning models to extract higher-order features or patterns in dimensions beyond 1D space.

Despite the promise of utilizing image-based deep learning models for genome analysis, there are challenges to be addressed. While genomic data can be converted into image input for analysis with deep learning models, it’s important to be aware of the different data characteristics. Genomic data is typically categorical, and the commonly used categorical representation lacks smoothness, presenting challenges for training deep learning models [79]. On the other hand, the interpretation of results from deep learning models can be complex, and there is a need for more transparent and interpretable models. Additionally, the computational demands of training and applying these models, particularly on large genomic datasets, are significant.

Looking ahead, image visualization for genome analysis is moving towards the development of more efficient, interpretable, and scalable deep learning models for genome analysis. There is also an increasing focus on integrating multi-omics data, combining genomic, transcriptomic, and proteomic information, to provide a more holistic view of biological systems. Genome analysis through image processing can provide a promising solution to achieve this goal.
